# Biosimilars as a Future, Promising Solution for Financial Toxicity: A Review with Emphasis on Bevacizumab

**DOI:** 10.7759/cureus.9300

**Published:** 2020-07-20

**Authors:** Tabinda Saleem, Hafiz Qurashi, Munira Jamali, Janet Chan Gomez, Tejaswi Kanderi

**Affiliations:** 1 Internal Medicine, University of Pittsburgh Medical Center (UPMC) Pinnacle, Harrisburg, USA; 2 Internal Medicine, Dow Medical College, Dow University of Health Sciences (DUHS), Karachi, PAK

**Keywords:** biosimilars, oncologic application, bevacizumab, cost effective therapy, commercialization, global oncology costs

## Abstract

A biosimilar is a biochemical product like another already approved biologic agent, known as the reference agent. To be endorsed by the Food and Drug Administration (FDA), biosimilars must demonstrate that they are as safe and effective as their reference item, with no clinical distinction. Humanized monoclonal antibodies (mAb) are revolutionizing the treatment of gastrointestinal and gynecologic malignancies. Bevacizumab, trastuzumab, cetuximab, rituximab, and pegfilgrastim are the most widely used mAb products with oncologic indications. Due to the complexities of the regulatory system, it may take time for anti-cancer biosimilars to play a significant game-changing role. Over the last decade, the use of generics has saved billions of dollars every year, and it is expected that biosimilars will soon prove to be a cost-effective alternative and can play an important role in driving down healthcare costs globally.

In this review, we provide a critical appraisal of biosimilars with an emphasis on bevacizumab-awwb (Avastin) and its clinico-pharmacologic characteristics, safety, efficacy, interchangeability, regulatory and oncologic perspectives, and overall clinical perception.

## Introduction and background

A biosimilar is a biochemical with a composition similar to a reference biologic agent and is approved for use in the United States (U.S.) by the Food and Drug Administration (FDA) [1]. For a product to be rendered as a biosimilar, there should be no clinically significant differences with its reference biologic agent. Apart from minor contrasts, the safety, efficacy, potency, and immunogenicity of the two should remain indistinguishable [2].

For a decade, biosimilars have been a fundamental part of clinical practice in the European Union. In 2006, somatropin (recombinant human growth hormone) was the first biosimilar introduced by the European Medicines Agency (EMA), followed by biosimilars for epoetin alfa and filgrastim over the next few years [3]. Furthermore, the introduction of biosimilar adalimumab in 2017 has led to an almost 80% reduction in the use of biologic adalimumab (Humira). Till now, a total of 16 biosimilars are available in the European market and many more are reported to be under process. On the other hand, in the United States, the FDA has approved 17 biosimilars so far but only seven are commercially available with filgrastim-sndz (Zarxiom) being the first one [4].

Currently, the pioneer manufacturers of biosimilars are in the United States, Europe, and Israel, whereas India, China, and Brazil have emerging manufacturers [5-6]. Many of the oncologic biologics have lost or are in danger of losing their patent protection, and to overcome this loss, new biosimilars are being developed [7-9]. The driving force behind the rapid advancement and introduction of biosimilars into the market is to increase the availability of cost-effective alternatives to biologics and thereby decrease overall health care costs, inciting the authorities to have a more optimistic impression on the biosimilars. In fact, as part of the current administration's efforts to lower drug prices, the FDA has released plans for the easier approval and marketing of biosimilars in the past; nonetheless, disputes between the makers of biologic drugs and biosimilar manufacturers are cited to cause delays in the actual implementation.

## Review

Out of the seven biosimilars approved for use in the United States, Zarxiom (filgrastim-sndz), a biosimilar to the granulocyte stimulating factor, filgrastim, was the first biosimilar to be approved in 2015, Please refer to Table 1 [10]. Additionally, pegfilgrastim-jmdb and the pegfilgrastim Lapelga (approved in Canada) are in the process of undergoing the administrative audit process [11-13]. As of late 2019, 25 biosimilars have been affirmed in the European Union (EU). Of them, none have demonstrated any distinction in the safety, adequacy, or frequency of adverse responses with the respective reference biologics when monitored over the course of 10 years. Whereas in the United States, as of late 2019, three biosimilar mAbs have been endorsed and are widely used; interestingly, their use is limited to chronic inflammatory conditions like rheumatoid arthritis, psoriatic arthritis, inflammatory bowel disease, etc., instead of oncologic indications. Infliximab-dyyb, a biosimilar of the tumor necrosis factor-alpha (TNF-alpha) inhibitor, Infliximab, is the first in this class and is the second biosimilar in the U.S. to get FDA endorsement [14]. Three ancillary TNF-alpha inhibitor biosimilars, etanercept-szzs, adalimumab-atto, and infliximab-abda, have FDA endorsement but are pending accessibility in the United States due to patent rights [15-17].

**Table 1:** A summary list of the biosimilars currently affirmed in the United States and their proposed indications Source: [18] FDA: U.S. Food and Drug Administration; HER2: human epidermal growth factor receptor 2

Biosimilar	Reference Drug	FDA Approval Timeline	Mechanism of Action(s)	Clinical Indications
Filgrastim-sndz1 (Zarxio)	Filgrastim (Neupogen/ Amgen)	2015	Granulocyte colony-stimulating factor	Acute myeloid leukemia, Severe neutropenia, patient on chronic immunosuppressive therapy, or those undergoing stem cell or bone marrow transplant.
Infliximab-dyyb (Inflectra)	Infliximab (Remicade)	2016	Tumor necrosis factor-alpha inhibitor	Juvenile idiopathic arthritis, Rheumatoid arthritis, Inflammatory bowel disease (Crohn’s disease or ulcerative colitis), and seronegative spondyloarthropathies.
Etanercept-szzs (Erelzi)	Etanercept (Enbrel)	2016	Tumor necrosis factor-alpha inhibitor	Severe active psoriatic disease, juvenile idiopathic arthritis, severe polyarticular juvenile idiopathic disease.
Adalimumab-atto (Amjevita)	Adalimumab (Humira)	2016	Tumor necrosis factor-alpha inhibitor	Rheumatoid arthritis, severe seronegative polyarticular disease, and inflammatory bowel disease
Infliximab-abda (Renflexis)	Infliximab (Remicade)	2017	Tumor necrosis factor-alpha inhibitor	Inflammatory bowel disease, rheumatoid arthritis, seronegative spondyloarthropathies.
Trastuzumab-dkst (Ogivri)	Trastuzumab (Herceptin)	2017	HER2 receptor inhibitor	HER2 receptor-positive metastatic breast disease, gastro-esophageal junction metastatic disease.
Epoetin Alfa-epbx (Retacrit)	Epoetin Alfa (Epogen/ Procrit)	2018	Erythropoietin	Anemia, cancer, chronic kidney failure
Pegfilgrastim-jmdb (Fulphilia)	Pegfilgrastim (Neulasta)	2018	Granulocyte colony-stimulating factor	Decrease the risk of infection in non-myeloid cancer who are receiving myelosuppressive chemotherapy
Filgrastim-aafi (Nivestym)	Filgrastim (Neupogen)	2018	Leukocyte growth factor	Reduce the frequency of febrile neutropenia and infections in patients with non-myeloid malignancies receiving immune-suppressive therapy.

Biosimilars for a complex biologic monoclonal antibody (mAbs) that are extensively utilized in the management of cancers (like trastuzumab, bevacizumab, rituximab, and cetuximab) are in the late phases of clinical advancement and are demonstrating comparative clinical efficacy to their reference [19-20]. Please refer to Table 2.

**Table 2:** A summary of oncology biosimilars in phase III trial in the United States includes either completed or underway products Source: [18] VEGF: vascular endothelial growth factor; HER2: human epidermal growth factor receptor 2; CD: cluster differentiation; EGFR: endothelial growth factor receptors; GCSF: granulocyte colony-stimulating factor; NSCLC: non-small-cell lung cancer; DLBCL: diffuse large B cell lymphoma; NHL: non-Hodgkin lymphoma

Product Name	Biosimilar	Class / Mechanism of Action(s)	Current Status	Clinical Indication
Bevacizumab	BCD-021 ABP 215 CT-P16 TAB008	VEGF inhibitor	Phase III initial trial approved for CT-P16	Colorectal metastatic disease, NSCLC, renal cell metastatic disease, glioblastoma, epithelial cervical, ovarian, and fallopian tube cancers.
Trastuzumab	Myl-1401O ABP 980 CT-P6 BCD-022 SB3	HER2 inhibitor	Phase III trial CT-P6 exhibited proportionate viability, safety; FDA endorsement pending	HER2-positive metastatic breast cancer.
Rituximab	GP2013 BCD-020 CT-P10 RTXM83	CD20 inhibitor	BCD-020 and RTXM83 demonstrated equivalent efficacy & safety in phase III trial; Others Listed/ ongoing FDA review.	Follicular lymphoma, DLBCL and NHL
Cetuximab	ABP 494 STI-001	EGFR inhibitor	ABP494 in phase III trial	Colorectal cancer
Pegfilgrastim	LA-EP2006 CHS-1701 MYL-1401H Lapelga	Pegylated G-CSF	MYL1401H, CHS-1701 acknowledged for FDA assessment.	Long-acting formulation of Filgrastim. Reduce the frequency of infections in patients compelling myelosuppressive therapy

Bevacizumab-awwb

**Figure 1:**
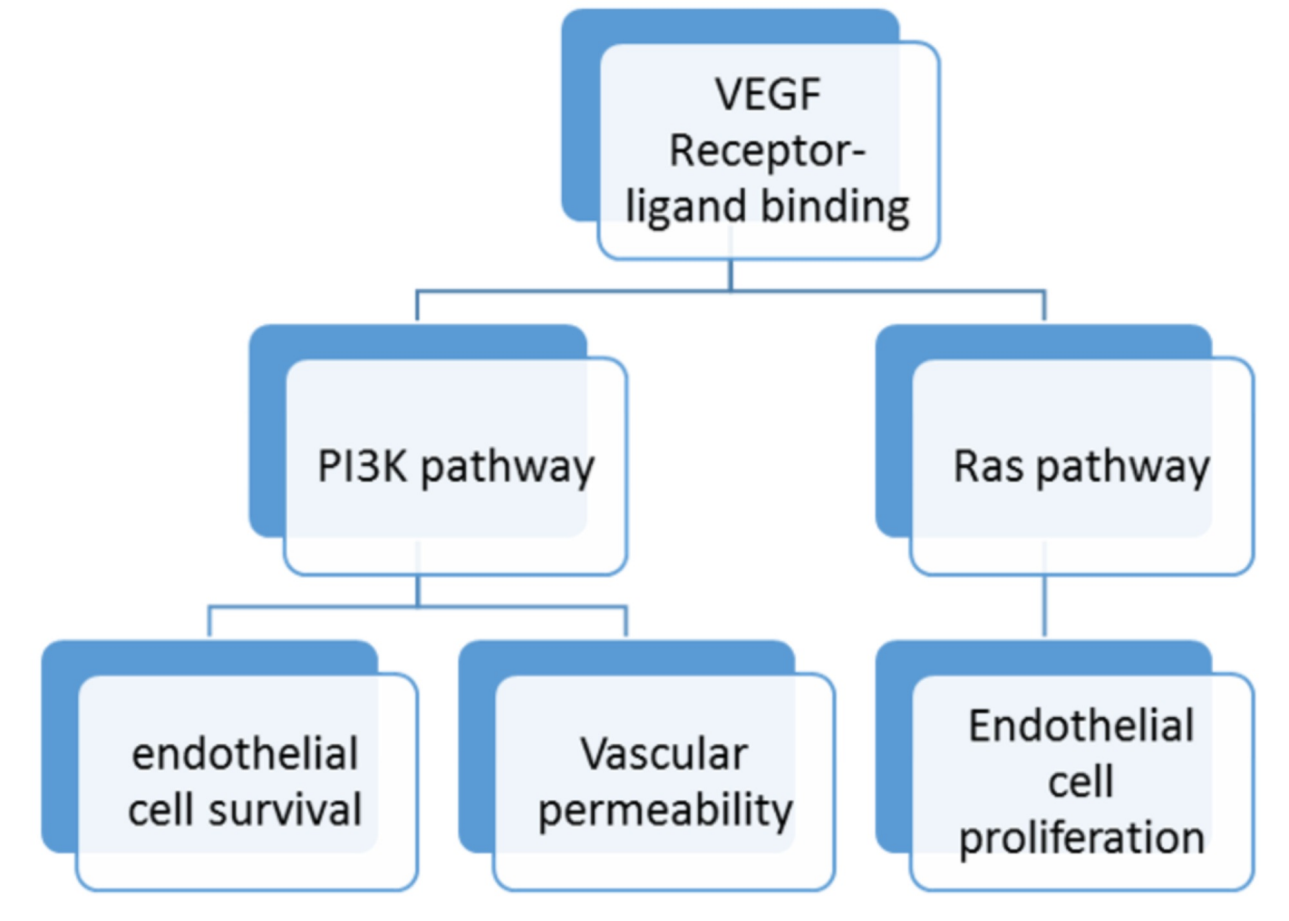
Schematic representation of the VEGF ligand and receptor binding Abbreviations: VEGF = vascular endothelial growth factor; PI3K = phosphatidylinositol-4,5-bisphosphate 3-kinase

Bevacizumab is a recombinant humanized monoclonal (IgG1) antibody that binds the vascular endothelial growth factor (VEGF) and prevents its interaction with VEGF receptor-1, and VEGF receptor-2 on the endothelial cell surface, resulting in angiogenesis inhibition preventing the growth and development of tumors as represented by Figure 1.

Bevacizumab (Avastin) was first approved by the FDA in February 2004 as the first-line treatment for metastatic colorectal carcinoma in combination with chemotherapy, which later gained popularity as first-line therapy for advanced non-squamous non-small cell, non-estimated glomerular filtration rate (eGFR) mutant lung carcinoma. Avastin was also approved for second-line therapy for glioblastoma multiforme and renal cell carcinoma in 2009. Since then, many randomized clinical trials have shown increased survival benefits making it one of the most popular oncologic drugs. A study published in 2014 showed good responses in various gynecological malignancies [21]. Please refer to Table 3 for indications of bevacizumab.

**Table 3:** Bevacizumab indications VEGF: vascular endothelial growth factor

FDA Approved Indications	Combination Chemotherapy	Type of Cancer	Indication for Second-Line Treatment
First-line treatment	Intravenous 5-fluorouracil	Colorectal metastatic disease	Patients who have progressive disease despite maximum therapy with bevacizumab.
	Fluoropyrimidine-irinotecan or fluoropyrimidine-oxaliplatin		
First-line treatment	Carboplatin and paclitaxel	Advanced, unresectable or recurrent non-small cell lung cancer	Patients with metastatic brain disease or on anticoagulant therapy
First-line treatment	Interferon-alpha	Renal cell metastatic disease	Nonresponder to initial therapy with other VEGF inhibitors
First-line treatment	Topotecan and paclitaxel or cisplatin and paclitaxel	Advanced, recurrent or persistent cervical cancer	To improve median survival with first-line chemotherapy
Second-line treatment	Monotherapy	Glioblastoma	Indicated for resistant and progressive disease in adult patients

FDA endorsed bevacizumab-awwb (ABP215 or MvasiTM), a biosimilar, in 2017 and since then it has been reported as an equally effective alternative to bevacizumab for treating various kinds of malignancies. Apsangikar et al. observed that biosimilar bevacizumab-awwb was non-inferior to the reference drug in treating metastatic colorectal cancer [22].

Similar outcomes are also reported between ABP-125 and the bevacizumab reference product in the treatment of advanced non-small-cell lung cancer in a phase III trial (the MAPLE study), conducted in Canada from 2015-2018 [23]. This randomized, double-blind, phase III comparative trial showed a response rate risk ratio of 0.93 with a 90% confidence interval (CI) between 0.80-1.09, which was similar for both Avastin and bevacizumab ABP-125 meeting the primary endpoint [23].

To date, many biosimilars of bevacizumab, with equal efficacy to Avastin, are reported to be in development globally. In a phase I clinical trial in China, bevacizumab biosimilars MIL 60, BAT1706, and IB1305 showed similar biochemical properties and biological functions. Pharmacokinetic (PK) profiles have a bioequivalence acceptance range of 80%-125% in comparison with the reference bevacizumab - EU.

In June 2018, a biopharmaceutical organization in South Korea, Celltrion, finished a phase I study on CT-P16's (biosimilar of bevacizumab) safety and pharmacokinetic evaluation. Clinical Trial Application (CTA) for phase III preliminary was presented to Portugal's National Authority of Medicines & Health Products, I.P. (Infarmed) with the idea to set phase III trials in 20 nations with more than 150 multi centers

In 2019, a phase I randomized a single-dose study conducted in China to evaluate the efficacy of TAB008 also showed similar pharmacokinetics and safety to Avastin.

Need for biosimilars

Biologics have become an integral part of many chemotherapeutic regimens. The spectrum of products, particularly the monoclonal antibodies, require a highly precise and expensive manufacturing process. The rising cost of cancer treatment (referred to as financial toxicity) and complex manufacturing processes limit their use, especially in developing countries. The expiration of patents for many biologics has paved the need for the development of corresponding biosimilars [1].

Biosimilars are novel structures that are planned to be profoundly like and treat similar conditions as a current biologic agent [24-25]. Biosimilars may offer expanded treatment alternatives for patients and physicians and can improve efficiencies across the healthcare system around the world. Biosimilars may also considerably bring down the cost of biologics and, consequently, increase the utilization of biologic treatments. This may further enhance clinical results and give more options to the prescribers, pharmaceuticals, and insurance companies and would overall abate the rising financial toxicity in the society.

Cost-effectiveness of biosimilars

**Figure 2:**
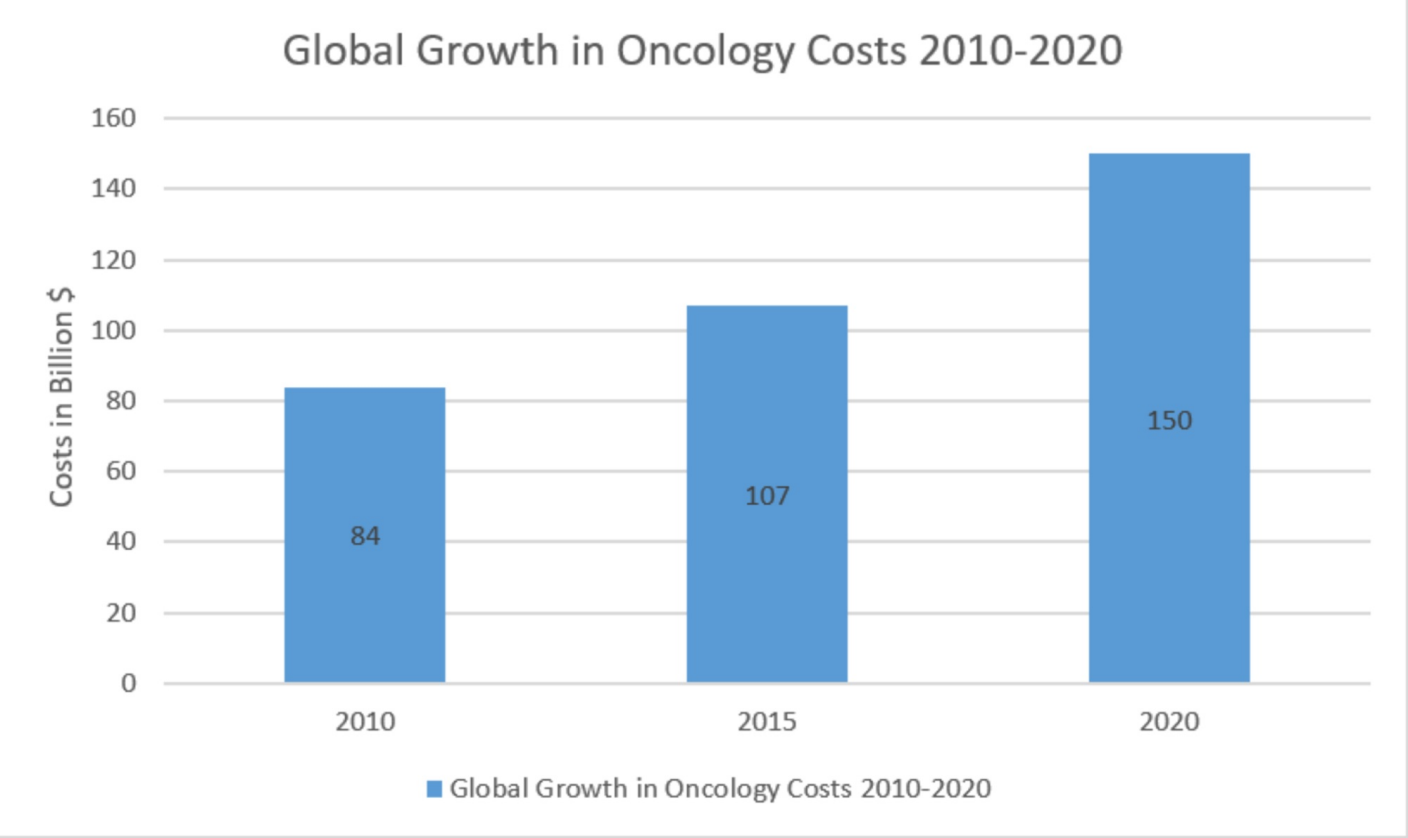
Graphical representation of rising global cancer drug expenditure by 2020 Source: The IMS (Intercontinental Marketing Statistics) Health (adapted from Lawlor 2016) [26]

The expanded use of biologic agents in cancer treatment is contributing to the continued rise in U.S. healthcare costs, with limited access. The Intercontinental Marketing Statistics (IMS) wellbeing report published in 2016 predicted that the global spending on oncology drugs will surpass $150 billion by 2020, with a yearly increase in the rate of up to 10.5% [26]. Please refer to Figure 2.

Since the establishment of the Biologics Price Competition and Innovation Act (BPCIA) in 2010, biosimilars have been created and showcased as aggressive, cost-effective alternatives to biologic medications [27-28]. Economists have noted that the accessibility of biosimilars may bring down the social insurance consumptions.

Eight of the 10 expensive medications currently available are used for cancer therapy. The monoclonal antibodies, bevacizumab, rituximab, and trastuzumab, are among the top-20 most expensive neoplastic drugs used in outpatient cancer centers, with the assessed worldwide expenditure of $5.6 billion for bevacizumab, $5.1 billion for trastuzumab, and $7.5 billion for infliximab [29]. Although only 15% of the agents listed in the National Comprehensive Cancer Network (NCCN) Drugs and Biologics Compendium are biologic agents, they dominate the drug therapy expenditure in the U.S. [27]. The patent for every one of these medications will expire within the next five years, thus necessitating the need for biosimilars, with encouraging results with biosimilars for bevacizumab. Expanding the use of these agents can lead to a significant decrease in the expense of cancer treatment, which eventually would reduce the insurance costs and co-pays for patients. However, the window for these biosimilars to have an impact on healthcare costs is narrowing owing to delays in market introduction and utilization. Advanced, more expensive treatments are taking the place of current biologics, lessening the financial impact of current biosimilars [30]. For biosimilars to cause significant flattening of the cost-expenditure curve, strong acknowledgment of their efficacy is needed by oncologists and their patients globally.

## Conclusions

We believe biosimilars have vast opportunities in the future, but bringing them to a level that ensures wide availability in the market is a long road ahead considering the costs. However, now that the key stakeholders are starting to better understand and accept biosimilars, it can result in more tolerant regulations and legislations that can ultimately lead to an increase in the utilization of biosimilars in the United States. We strongly believe that understanding the concept of biosimilars is crucial for oncologists.
